# The Institutional Worker

**Published:** 1912-12-07

**Authors:** 


### THE INSTITUTIONAL WORKER

**Being a Special Supplement to "The Hospital"**

### BUREAU OF INFORMATION.

### Rules for Correspondents.

1. Every letter, on whatever subject, must be accompanied by the coupon to be cut from the back cover (inside page) of The Hospital, current issue, and must contain the name and address of the correspondent with pseudonym for publication if desired.

2. Replies by post cannot be given, save under exceptional circumstances at the Editor's discretion.

3. Letters from Approved Homes in reply to special needs published in the Bureau should state terms and full particulars, and be sent prepaid under cover to the Editor of the Bureau with name written across coupon for identification.

4. Proprietors of Homes which have not yet been entered on the List of Approved Homes, but have spare accommodation likely to suit special needs, are invited to write for an application form for registration. The fee for registration, which includes two announcements of the Home in the Bureau, is 5s.

5. Special needs of every description relating to those who are sick or in difficulties are made known in these columns free of charge.

6. All communications to be addressed to The Editor of The Hospital, 28 Southampton Street, Strand, London, W.C., and marked "Bureau of Information."

### Approved Homes.

Note.—*No Home is added to the List without direct medical and other testimony to its good management.*

**Hemel Hempstead.**— Bracken Lodge, 44 Marlowes. Principals: The Misses Eastty. Medical, surgical, midwifery, and convalescent cases. Scalp massage. Special reduction for necessitous nurses. Bracing climate.

### Needs and Helpers.

**Blind and Deaf.**—We are very happy to learn that you have procured another subscriber to add to the one enlisted by the Bureau, and that you can now be nominated for a pension from the National Blind Relief Society. You will see by their regulations that the Hon. Secretary cannot guarantee an immediate pension, but within a year at the outside you will have 5s. a month, with the prospect of succeeding to one of 10s. a month in due course. Will you tell your friends that we are trying to get together a little sum to provide you with the 5s. a month until the pension begins to come due? Ask them what they can do.—J. B., Maidstone.

**Electric Treatment for Rheumatic Patient.**—As the patient is unable to pay for treatment, apply to a hospital with good electric equipment, such as the London or the Middlesex. Send medical certificate and letter explaining the circumstances.—Hope (139x).

### Homes Wanted.

**Sanatorium for Young Lady.**—The patient is twenty years of age, with a slight affection of one lung, and has been advised that she ought to have sanatorium treatment for at least a couple of months. She is dependent on her sister, who is a nurse, and cannot afford high terms. A Home which offers conditions on the regular lines of open-air treatment is desired. Replies, stating lowest terms, to J. M. J. (140a).

**Home for Asthmatic Patient.** — A lady, who is suffering from bronchial asthma, wishes to find a suitable Home in which to spend the winter and spring. The doctors have suggested going abroad, but her means will not allow of such an expense.—Katrina (141a).

**For Invalid Superintendent.**—We trust one of our Homes has been able to receive this patient. If not, write to the Home for Confirmed Invalids, 36 Aubert Park, Highbury, N., where, if there is a vacancy, she could be received for 15s. a week. Thank you for your appreciation of the Bureau as supplying "a much-needed source of mutual help to homes and patients."—K. M. L. (135a).

### Nursing in the Empire and Abroad.

**Conditions in Bloemfontein.**—The climate is dry and healthy, and the place is a resort for invalids. Write to the Secretary (Mr. Doel), the National Hospital, Bloemfontein, or to Dr. Dellacott, consulting surgeon, The Jagersfontein Mine Hospital, S.A. You seem to be well qualified for work in the Colony except in midwifery, and we think if you want to go out it would be worth your while to take a course of this first. You could probably command higher fees in Bloemfontein than in British Columbia, as conditions are more settled. We are advised that the demand is small for nurses in the latter locality, except in the wilds, where the conditions of work are very primitive.—E. T.

**A Need in Montevideo.**—We understand that one or two trained nurses could do very well if they made the venture, as there are none out here. The Matron of the British Hospital, Camino Aldea, Montevideo, would give you full information, should you decide on taking up work in the town. Midwifery is essential.—Ell.

**Nursing in Bangkok.**—The Bangkok Nursing Home employs nurses who are sent out by the Colonial Nursing Association. There is no demand for private nurses whatever. Your right course would be to apply to the Colonial Nursing Association.—Esther V.

### Letter-Box.

**The Editor begs to acknowledge the receipt of communications, with enclosures, from:**—Mrs. E. W.; Miss M. E. G.

**Communications have been received and attended to from:**—Miss H. D. P., Clare; M. E., Llangollen; M. B., W. Croydon; Miss L. R. H., Nottingham.

**The Editor is much indebted for testimonials in response to inquiries from:**—Dr. G., Wimbledon Rev. E. R. T., Altrincham; Dr. H., Boxmoor; Dr. A. G., Altrincham.

**Letters have been forwarded from:**—Mrs. G. Mrs. D.; Miss M.; Miss K. J. W., Westcliff; Mrs. A.; Sister A.; Miss McC.; Miss E. A. M.; Miss M. R.; Mrs. G., Worthing (coupon omitted, please remember it another time).

E. P., Clacton; M. E., Llangollen; R. S.; Mrs. B., Winton.—We are unable to introduce patients to any but the Homes on our Approved List.

F. W. P.—All the Homes we have named are quite to be relied on.

H.B.M. Vice-Consul, Montevideo.—Many thanks for the trouble you have taken for us.

R. E. M. (133a).—Very glad one of our Homes has been able to receive your friend.

H.B.M. Vice-Consul, Bahia Blanca.—Many thanks. We note there is little demand for nurses in your locality.

**Persons requiring nurses and nurses requiring appointments will find their needs met in the adjoining pages, where many useful announcements appear this week.**

### INSTITUTIONAL HOUSEKEEPING.

### Readers' Questions with Practical Answers.

**Peeling Potatoes in Advance.**—On no account allow your cook to have sufficient potatoes peeled to last two days, for after being peeled they must, of course, be immersed in cold water to prevent discolouration. Potatoes contain a small percentage of salts, and these saline constituents are most valuable to health. Potash salts are disseminated through the potato, and are freely soluble in water. Thus potatoes deprived of the skin, which would resist the passage of the potash into the water, lose most of it, and their food value is greatly lessened. It is this reason also which renders the steaming, or cooking of potatoes in their "jackets," so desirable, especially for those who obtain only a small amount of raw salads, fruits, and other vegetables.—N. P. (Shropshire).

**Dishes Without Meat — Savoury Rice.** — Well wash 14 lb. of rice, throw it into a large boiler containing boiling salted water, add threepennyworth of coarsely-chopped onions, and boil these until the rice is soft. Then strain out these two ingredients, or drain off all the water very thoroughly. Add 3 lb. of grated stale cheese (it is a good way of utilising scraps), 1 lb. of good dripping, pepper, salt, and stiffly mixed mustard to taste. Heat thoroughly piled up in vegetable dishes. As, of course, your materials will be purchased in large quantities, this amount should cost about 4s. 6d. and be sufficient for fifty persons.—SUPERINTENDENT.

**Burst Pipes during the Winter.**—Your troubles every winter arise entirely from want of foresight. Long before the cold weather sets in, protect all exposed pipes, taps, and cisterns with bands of straw, felt matting, or any thick material. If they are inaccessible, get a plumber to pack them. When a hard frost is in progress keep a lamp burning in the room where the cistern is placed, or leave gas jet partly on. The trifling expense will be more than compensated by freedom from burst pipes.—E. G. M.

**Diet Scheme for Patients Forbidden Meat.**—Your difficulty is one which is often experienced by the principals of nursing homes, for it is by no means uncommon for the consumption of meat and poultry to be forbidden or temporarily suspended, and it is not everyone who fancies a fish diet. We are, therefore, devoting considerable space to answer this problem for you. The following specimens of vegetarian meals are planned to contain as far as possible the right food values; they mostly contain eggs, milk, and cheese, and will be enjoyed by all as a change from animal food.

BREAKFAST.—Oatmeal or hominy porridge may be served, followed by eggs cooked in various ways, fried bananas, tomatoes or potatoes. Fresh or stewed fruit, jam or marmalade, should accompany the meal to supply sugar.

DINNER.—(1) Cheese soup, lentil rissoles and tomatoes, potato saunders, gingerbread pudding and sauce. (2) Mock white fish, Scotch stew, macaroni cheese, nut sandwiches.

SUPPER.—Digestible foods must be chosen, and too much cheese must therefore be avoided. Such dishes as a savoury semolina pudding served with tomatoes, or a stew made with all kinds of vegetables and haricot beans will be nourishing and light; fruit should follow in some form.

The foregoing suggestions are in season all the year round, but in summer the addition of much fresh fruit, lettuce, and salads of all kinds adds greatly to the delight of the menu.

RECIPES FOR ABOVE DISHES.

CHEESE SOUP.—2 oz. cheese, 1 oz. butter, ½ pint milk, 1 egg, 1½ pint water, 1 small onion, seasoning, a French roll. Cut the roll in pieces, dry in the oven. Heat the butter in a stew-pan. Fry the sliced onion. Add milk and water. Boil up. Add cheese. Draw aside. Add the egg beaten. Season well. Cook till it thickens; *it must not boil.* Put the bread in a tureen. Pour over the soup. *Cost* for six persons, 6d.

LENTIL RISSOLES AND TOMATOES.—4 oz. lentils, ½ oz. butter, seasoning, short crust paste. Cook the lentils in boiling water till tender. Mash with the butter and seasoning. Roll the paste very thin, cut into rounds. Put a little mixture on each. Damp. Fold up, egg and breadcrumb. Fry in oil in deep pan. Serve hot with baked tomatoes. *Cost*, 3d.

POTATO SAUNDERS.—Mashed potato, seasoning, chopped parsley and herbs, bread crusts soaked in water, chopped fried onion. Roll out the mashed potato, cut into squares. Put on each a mixture of bread, herbs, parsley, onion, and seasoning. Fold up like a sausage-roll. Bake till firm and browned.

MOCK WHITE FISH.—Salsify, white sauce, breadcrumbs, lemon-juice. Scrape the salisfy, cut the roots into 2-inch pieces. Cover with lemon-juice and water. Soak one hour. Drain. Cook gently in boiling salted water till tender. Place in cockle-shells. Cover with the white sauce well seasoned, and sprinkle thickly with breadcrumbs. Add a few pieces of butter. Brown in the oven. *Cost*, 3d. for three persons.

SCOTCH STEW.—Carrot, turnip, onion, celery, half a cabbage, some haricot beans, 2 oz. pearl barley, seasoning. Blanch the barley. Simmer with the beans ¾ hour in fresh water. Cook all the vegetables cut into large pieces (omit the cabbage) in enough boiling water to cover. Add the barley and beans after the first ten minutes, and the seasoning. Shred the cabbage finely; add when the vegetables are nearly done. Cook for another ten minutes, and serve hot. *Cost*, 7d. for six persons.

NUT SANDWICHES.—Grind any kind of nuts in a nut-mill. Season with pepper and salt. Spread between brown bread and butter. Decorate with parsley.

INEXPERIENTIA.

ALMOND ICING.—1 lb. ground almonds, ½ lb. icing sugar, ½ lb. castor sugar, 1 whole egg and 1 yolk, juice of 1 or 2 lemons, flavouring to taste. Sieve the icing sugar. Pound all ingredients together.

ROYAL ICING.—1 lb. icing sugar, 2 whites of eggs, juice of 1 lemon. Sieve the sugar. Slightly beat the whites. Add the sugar gradually. Beat very thoroughly. Add the lemon-juice to keep it white. Always keep covered unless the beating is being done. Use for coating and decoration, and apply with icing funnel.—ALBERTA.

---

NOTICE TO OUR READERS.

The Editor welcomes for Institutional Housekeeping any contribution from those engaged in the work of the commissariat in institutions. All accepted contributions are paid for. Queries answered on any subject relating to the supply, cost, storage, distribution, or preparation of food.

### Editor's Notices.

**Contributions:**

Contributions should be written, or preferably typed, on one side of the paper only, and all articles sent in are accepted upon the distinct understanding that they are forwarded to The Hospital only.

The Editor cannot undertake to return MSS. not used, but when a stamped directed envelope is enclosed the MSS. may be returned if a special request is made.

Accepted articles and paragraphs of news will be paid for after publication at the scale rate.

**Address:**

To prevent delay all contributions and letters on editorial business must be addressed exclusively to the Editor, "The Hospital," 29 Southampton Street, Strand, London, W.C. It is important that this regulation shall be strictly observed.

**Correspondence:**

Correspondence on all subjects is invited. The name and address of correspondents must be given as a guarantee of good faith, but not necessarily for publication.

**Special Articles:**

Special articles are invited, and questions, inquiries, and paragraphs upon all matters relating to the work, administration, and management of general, special, mental, fever, cottage and convalescent hospitals, sanatoria, homes, institutions, societies and organisations for the treatment or care of the sick, injured and dependants of all classes. Every matter affecting the interests and welfare of the staff of all grades working in these institutions will receive special consideration.

**Administrative Medicine, Research and Items of News:**

Special terms will be paid for approved articles on subjects in this wide field by experts who have made some section of it their special study and interest. We wish to give prominence to every movement tending to advance accuracy of diagnosis, efficiency in treatment, and the development of modern methods to eradicate disease and promote the welfare of the suffering.

**A Bureau of Information:**

We invite inquiries and applications for information and help in providing for that numerous class of sufferers who require special care or house-room which they cannot obtain in their own homes or secure for themselves. **We cannot, however, prescribe, or recommend practitioners.**

**Books for Review:**

Publishers are particularly requested to send advance proofs of any new books of importance whenever possible, as the Editor has made arrangements to publish immediate reviews on a new plan.

**Photographs, Plans, Blocks, and Illustrations:**

It is requested that wherever possible MSS. may be accompanied by illustrations in any of the above forms. The name of the sender and of the article to which it belongs should be written on the back of each photograph, drawing, or block for purposes of identification.

**Local Papers:**

Newspapers containing reports or news paragraphs should be marked and addressed to the Sub-Editor.

**The Coupon System:**

A coupon will be found at the bottom of the third inside page of the cover each week. This coupon must be attached to every question or inquiry to which an answer is desired in The Hospital.

### You Should Send for the Booklet containing particulars of The Hospital Bureau of Information.

The purpose of this Department is to become a practical help to *every* Institutional Worker, Health Visitor, District Nurse, and all associated with the Prevention of Disease, Care of the Sick, and the Advancement of National Health.

The want of accurate information on the many phases of Institutional Work has been the stumbling-block in the past, but with the inauguration of The Hospital Bureau of Information this need be no longer a bar to progress.

Years of experience in Institutional Affairs and the Medical Health and Sanitary Services are placed at the disposal of every reader of The Hospital requiring assistance *entirely without charge.*

A knowledge of the scope and work of the Bureau is a matter that deeply concerns your Professional career, and it is to your interest to obtain at once details of this new development.

All letters in regard to the Bureau must be addressed to The Editor, The Hospital Bureau of Information, The Hospital Building, 28 and 29 Southampton Street, Strand, London, W.C.

### Manager's Notices.

**All letters on business matters must be addressed to The Manager, "The Hospital," 28 and 29 Southampton Street, London, W.C.**

**Advertisements:**

To ensure insertion, all advertisements for The Hospital must reach the Manager not later than Wednesday at noon in each week. For scale of charges see pages vii and 4.

**Subscriptions,** which may commence at any time, are payable in advance. Cheques and Post-Office Orders should be crossed London County and Westminster Bank, Covent Garden Branch, and made payable to the Manager of The Hospital.

Rates of Subscription (including Postage).

| *United Kingdom.* | | *Foreign and Colonial.* | |
|---|---|---|---|
| Three Months ... ... | 2s. 0d. | Three Months ... ... | 2s. 6d. |
| Six Months ... ... | 4s. 0d. | Six Months ... ... | 5s. 0d. |
| Twelve Months ... ... | 6s. 6d | Twelve Months ... ... | 8s. 8d. |

*SUBSCRIPTION FORM.*

*Please send me* The Hospital *for..............months, commencing with your issue of.........................., for which please find enclosed...........................*

*Name*..............................................

*Address* ..............................................

..............................................

*Date*..............................

*To* .............................. *Newsagent.*

Or to

*The Manager,*
The Hospital,
The Hospital Building,
28/29 Southampton Street,
Strand, London, W.C.

**Remittances:**

**It is especially requested that remittances be made by Cheque or Postal Order, and in halfpenny stamps only when the amount is under sixpence.**

### BOOKS FOR INSTITUTIONAL WORKERS.

A Legal Handbook for the Use of Hospital Authorities 2s. 6d.

The District Nursing Association: Hints on How to Start It . . . . . . . . . . . 1s.

The Matron: Her Duties and Responsibilities . . 2s. 6d.

The Nurses' Pronouncing Dictionary . . . . 2s.

Art of Feeding the Invalid . . . . . . . 1s. 6d.

Furnishing and Appliances of a Cottage Hospital . 6d.

Burdett's Hospitals and Charities . . . . 10s. 6d.

Case-papers, Charts, Ledgers, Account Books, National Insurance pamphlets and every kind of Institutional literature.

Of all Booksellers, or of THE SCIENTIFIC PRESS, Ltd., 28 & 29 Southampton Street, Strand, London, W.C.

